# The Degree of Joint Line Obliquity Change Is Not Associated with Patient Preference in Bilateral Posterior-Stabilized Total Knee Arthroplasties

**DOI:** 10.3390/jcm15051889

**Published:** 2026-03-02

**Authors:** Sang Jun Song, Young Kook Kim, Sae Heon Kim, Cheol Hee Park

**Affiliations:** 1 Department of Orthopaedic Surgery, Kyung Hee University College of Medicine, Kyung Hee University Medical Center, Seoul 02447, Republic of Korea; songsjun@khmc.or.kr (S.J.S.); heon0515@naver.com (S.H.K.); 2 Department of Orthopaedic Surgery, Seoul Sacred Heart General Hospital, Seoul 02488, Republic of Korea; clipom63@gmail.om

**Keywords:** knee, total knee arthroplasty, posterior stabilized, joint line obliquity, preference

## Abstract

**Objectives:** To evaluate patient reference in paired bilateral posterior stabilized (PS) total knee arthroplasties (TKAs) with significantly different degrees of joint line obliquity (JLO) change. **Methods:** A retrospective review was conducted on 128 patients who underwent paired bilateral PS TKAs, with greater and smaller JLO changes (G-ΔJLO and S-ΔJLO knees; a side-to-side difference in JLO change >3° between G-ΔJLO and S-ΔJLO knees). The mean follow-up period was 3.9 years (minimum 2 years). Radiographic changes in JLO were measured according to the Coronal Plane Alignment of the Knee (CPAK) classification. Maintenance of the CPAK type and JLO direction (apex distal, neutral, and apex proximal) was investigated. Clinically, the preferred TKA out of greater and smaller JLO changes was investigated. The Hospital for Special Surgery and Western Ontario and McMaster Universities Osteoarthritis Index were evaluated. **Results:** The average JLO change was 10.5° in the G-ΔJLO knees and 5.5° in the S-ΔJLO knees (*p* < 0.001). The CPAK type and JLO direction was better maintained in the S-ΔJLO knees (*p* < 0.001, respectively). Regarding preference, 40 patients (31.2%) were satisfied with bilateral TKAs without a specific preference, while 44 patients (34.3%) preferred TKAs of the G-ΔJLO knee and 44 patients (34.3%) preferred the S-ΔJLO knee (*p* = 1.000). No significant differences were found in the patient reported outcomes between the G- and S-ΔJLO knees. **Conclusions:** The degree of joint line obliquity change was not associated with patient preference in bilateral PS TKAs. JLO preservation may not be a critical determinant of patient preference in PS TKA.

## 1. Introduction

Preservation of joint line obliquity (JLO) has recently garnered attention as a potential factor improving postoperative outcomes after total knee arthroplasty (TKA) [1,2,3,4]. Previous studies have suggested that JLO preservation contributes to better restoration of native knee kinematics and enhances clinical outcomes [1,2,3,4]. However, these studies primarily investigated cruciate-retaining (CR) TKAs, which preserve the posterior cruciate ligament (PCL), an important structure for replicating native knee kinematics. In posterior-stabilized (PS) TKA, which involves removal of PCL and thereby limits the ability to replicate natural knee kinematics, the preservation of the JLO may have limited clinical impact [5,6,7].

Multiple factors influence clinical outcomes after TKA, among which preoperative expectations, psychological factors, pain sensitivity, and lifestyle inevitably vary from person to person [8,9,10]. Most studies analyzing the relationship between JLO preservation and clinical outcomes have not rigorously controlled for these factors [2,5,11,12,13]. Given this limitation, a more reliable assessment of the effects of JLO preservation can be achieved by analyzing patient preference in TKAs performed on paired knees using the same prosthesis but with significantly different degrees of JLO change.

This study aimed to evaluate patient preference in paired bilateral PS TKAs performed with the same prosthesis but with significantly different degrees of JLO change. Our hypothesis was that greater JLO change would not be inferior to smaller JLO change with respect to patient preference.

## 2. Materials and Methods

### 2.1. Patients

This retrospective study included patients who underwent 1-week staged bilateral primary TKA using either Attune^®^ (Depuy Synthes, Warsaw, IN, USA) or Persona^®^ (Zimmer, Warsaw, IN, USA) PS prosthesis at our hospital between January 2018 and December 2022. All surgeries were performed by a senior surgeon who had surgical experience with over 3000 TKAs before the study period.

**Figure 1:**
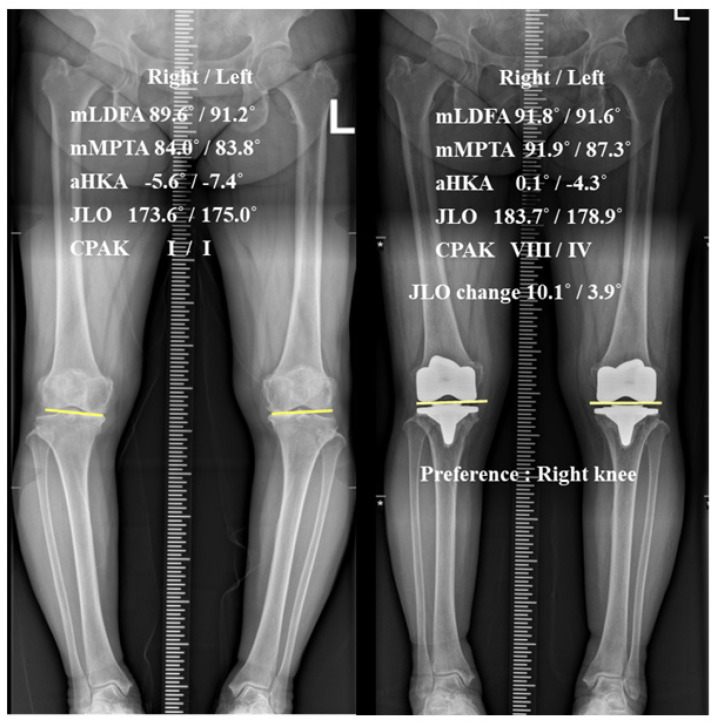
Bilateral paired total knee arthroplasties with greater and smaller changes in joint line obliquity (JLO).

**Figure 2:**
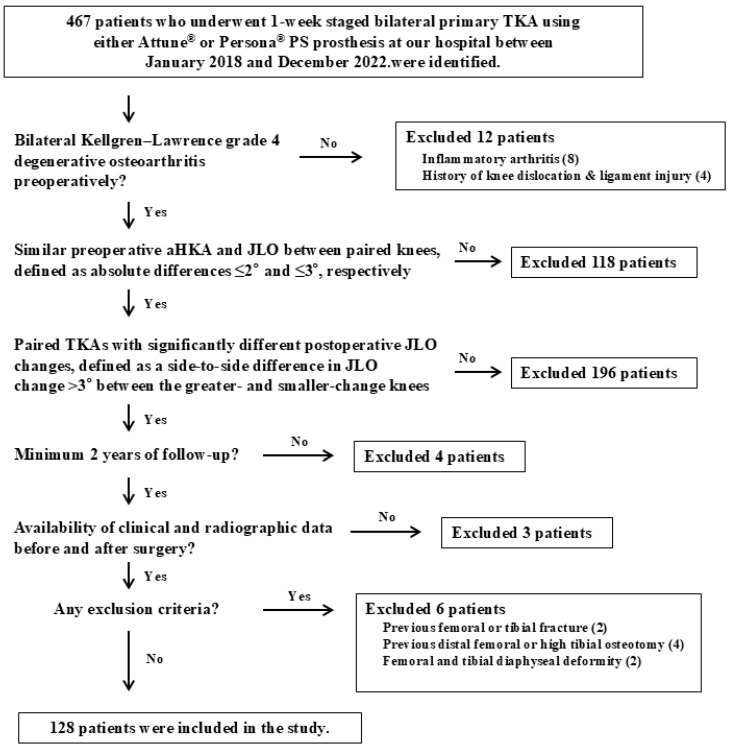
Flow chart. TKA, total knee arthroplasty; PS, posterior stabilized; aHKA, arithmetic hip–knee–ankle angle; JLO, joint line obliquity.

Patients were included if they met the following criteria: (1) bilateral Kellgren–Lawrence grade 4 degenerative osteoarthritis preoperatively; (2) similar preoperative arithmetic hip–knee–ankle angle (aHKA) and JLO between paired knees, defined as absolute differences ≤2° and ≤3°, respectively [14]; (3) paired TKAs with significantly different postoperative JLO changes, defined as a side-to-side difference in JLO change >3° between the greater- and smaller-change knees based on the previous literature [3]. Specifically, if JLO change, defined as postoperative JLO minus preoperative JLO, was A in one knee and B in the contralateral knee, the patient was included when |A − B| > 3° (Figure 1); (4) a minimum follow-up period of 2 years; and (5) availability of clinical and radiographic data before and after surgery. Exclusion criteria included: (1) inflammatory arthritis; (2) a history of femoral or tibial fracture, knee dislocation, or ligament injury; (3) a history of distal femoral or high tibial osteotomy; and (4) presence of femoral and tibial diaphyseal deformity. Based on these criteria, 128 patients were included in this study (Figure 2).

The patient preferred the right knee postoperatively, despite a greater JLO change in the right knee. The yellow line indicates the tangent line to the distal condyle of the native knee and femoral component, which serves as a reference for estimating JLO.

mLDFA, mechanical lateral distal femoral angle; mMPTA, mechanical medial proximal tibial angle; aHKA, arithmetic hip–knee–ankle angle; JLO, joint line obliquity; CPAK, Coronal Plane Alignment of the Knee; JLO change, postoperative JLO minus preoperative JLO.

The patient demographics are presented in Table 1. No significant differences were observed regarding preoperative clinical and radiographic knee conditions between paired TKAs with greater JLO change (Greater-ΔJLO knees) and smaller JLO change (Smaller-ΔJLO knees) (Table 2 and Table 3). This study was approved by the Institutional Review Board of our institution. Informed consent was obtained from all of the patients before commencing the review.

### 2.2. Surgical Techniques and Rehabilitation

All TKAs were performed using a tourniquet control. A medial parapatellar approach was used with a midline skin incision. An intramedullary guide was used for distal femoral resection. The distal femoral cut was performed at valgus angles of 4°, 5°, and 6° using the intramedullary rod as a reference, corresponding to preoperative long-leg radiographic measurements of the angle between the femoral anatomical and mechanical axes of <5°, 5–7°, and >7°, respectively. The transepicondylar axis was used for determining the femoral component rotation. An extramedullary guide was used for tibial resection, with the initial cutting plane positioned perpendicular to the mechanical tibial axis. The posterior tibial slope (PTS) was set at 3° in the sagittal plane, guided by a reference line connecting the fibular head and lateral malleolus. Tibial component rotation was determined using a reference line connecting the medial third of the tibial tubercle to the insertion point of the PCL.

Following resection, trial implants were inserted. Mediolateral and flexion–extension balancing was achieved through bony adjustment prior to any soft tissue release. For mediolateral balancing, modification of the tibial cutting plane was prioritized. The coronal alignment of the cutting plane was adjusted within the range of varus 3° to valgus 3°. Flexion–extension gap balancing was achieved by modifying the tibial slope within the range of 0° to 6° [3]. If imbalance persisted despite adjustments being made to the bony cutting plane, selective release of the contracted soft tissue was performed.

The appropriate thickness of the polyethylene insert was determined by considering stability and kinematics with proper ligament tension and physiological knee motion without hyperextension, flexion contracture, or lift-off of the trial insert. Patellar resurfacing was performed. All components were implanted on cleaned and dried cut surfaces using a full cementation technique.

Isometric exercises using the extensor and flexor muscles were initiated shortly after the operation. The drain was removed on the first postoperative day, followed by initiation of active and assisted range of motion (ROM) exercises. Weight-bearing ambulation was also initiated on the first operative day, as tolerated by the patients.

### 2.3. Radiographic Evaluation

The radiographic parameters were measured preoperatively and at the final follow-up visit. Preoperative and postoperative long-leg standing anteroposterior radiograph and lateral knee radiograph were obtained under weight-bearing conditions.

**Figure 3:**
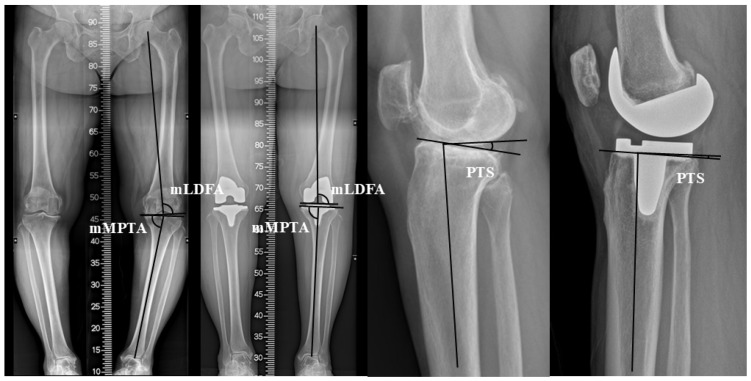
Radiographic measurements. mLDFA, mechanical lateral distal femoral angle; mMPTA, mechanical medial proximal tibial angle; PTS, posterior tibial slope.

The mechanical lateral distal femoral angle (mLDFA), mechanical medial proximal tibial angle (mMPTA), aHKA, and JLO were measured on long-leg standing anteroposterior radiographs in accordance with the Coronal Plane Alignment of the Knee (CPAK) classification system (Figure 3) [14]. mLDFA was defined as the lateral angle between the femoral mechanical axis and the tangent line of the most distal points of the femoral condyles in the native knee or implant. mMPTA was defined as the medial angle between the tibial mechanical axis and the tangent line of the native proximal tibial plateau or the tibial baseplate. aHKA was defined as the value obtained by subtracting mLDFA from mMPTA, whereas JLO was defined as the sum of mMPTA and mLDFA. PTS was measured using lateral knee radiographs [15]. Preoperative PTS was defined as the angle between the perpendicular line of the tibial intramedullary canal axis and the line connecting the anterior and posterior borders of the medial tibial plateau (Figure 3). For the measurement of the postoperative PTS, the tibial cutting surface was used (Figure 3).

Radiographic quality was ensured by standardizing knee positioning and maintaining a consistent distance between the X-ray beam and cassette. All images were digitally transferred to a Picture Archiving and Communication System (PACS). Angular measurements were performed using INFINITT PACS software (ver 7.0, INFINITT Healthcare, Seoul, Republic of Korea)., with a minimum detectable angular difference of 0.1°.

In order to minimize any observational bias, two independent investigators performed all radiographic measurements. The interobserver reliabilities of all measurements were assessed using the intraclass correlation coefficient, and all values were greater than 0.8. Therefore, the average values obtained by the two investigators were used for analysis.

### 2.4. Clinical Evaluation

The clinical outcomes were evaluated preoperatively and at the final follow-up visit. Patient preference between paired TKAs with greater and smaller JLO changes was investigated. The Hospital for Special Surgery score (HSS), Western Ontario and McMaster Universities Osteoarthritis Index (WOMAC), and ROM were evaluated. The ROM was measured using a long-armed goniometer.

### 2.5. Complications

Any complications were investigated according to the standardized list and definitions of complications provided by the Knee Society [16].

### 2.6. Statistical Analysis

The pre- and postoperative radiographic variables and clinical scores were compared between G- and S-ΔJLO knees using the paired *t*-tests. The proportion of CPAK type and JLO direction (apex distal, neutral, and apex proximal) were compared between the G- and S-ΔJLO knees using the Stuart–Maxwell test. The maintenance rates of CPAK type and JLO direction, as well as the preference rates, were compared using the McNemar test. All statistical analyses were performed using SPSS version 25.0 (Chicago, IL, USA). A *p*-value < 0.05 was considered statistically significant.

Power analysis was performed to determine the minimum sample size required for sufficient statistical power in evaluating patient preference. This analysis was based on a hypothesis of non-inferiority between G- and S-ΔJLO knees, with a clinically acceptable difference in preference rate set at 15% [17,18]. The alpha level was set at 0.05, and the desired power was 80%. The performed power analysis indicated that >119 patients were required for each group to ensure sufficient statistical power.

## 3. Results

Radiographically, no significant differences were observed in preoperative variables between the G- and S-ΔJLO knees (Table 2). Postoperatively, the femoral component showed more varus alignment in the G-ΔJLO knees, whereas the tibial component showed more varus alignment in the S-ΔJLO knees (Table 2). The average postoperative aHKA exhibited a more varus alignment in the S-ΔJLO knees; however, the mean change in aHKA between the two groups was not significantly different (Table 2). The average postoperative JLO displayed a more apex proximal obliquity in the G-ΔJLO knees (Table 2). The average JLO changes were 10.5° in the G-ΔJLO knees and 5.5° in the S-ΔJLO knees (*p* < 0.001). The preoperative and postoperative PTS and changes in PTS were not significantly different (Table 2).

**Figure 4:**
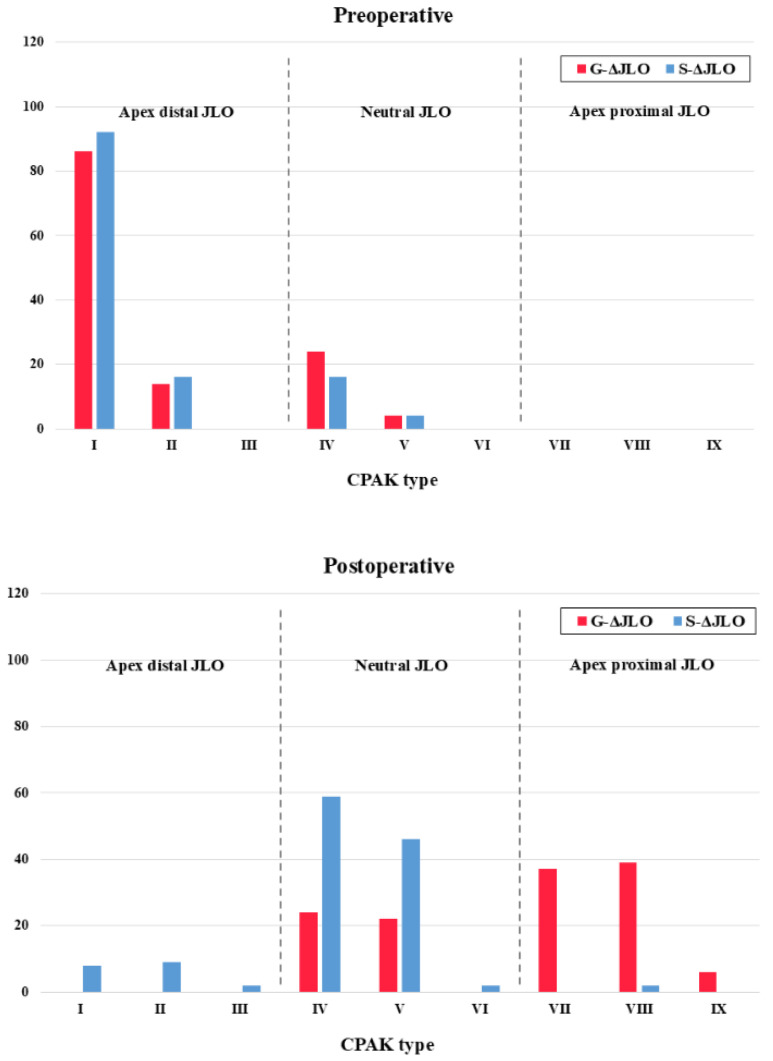
Preoperative and postoperative distribution of the Coronal Plane Alignment of the Knee types and direction of joint line obliquity. G-ΔJLO, TKAs with a greater change in the joint line obliquity; S-ΔJLO, TKAs with a smaller change in the joint line obliquity; CPAK, Coronal Plane Alignment of the Knee; JLO, joint line obliquity.

The postoperative proportions of CPAK type and JLO direction after surgery differed significantly (*p* < 0.001 for both) between the G- and S-ΔJLO knees, although the preoperative portions were not significantly different (*p* = 0.304 and 0.102, respectively) (Figure 4). Maintenance of the original CPAK type was observed in 0 cases in the G-ΔJLO knees and in 20 cases (15.6%) in the S-ΔJLO knees (*p* < 0.001). The direction of JLO was maintained in 0 cases in the G-ΔJLO knees and in 37 cases (28.9%) in the S-ΔJLO knees (*p* < 0.001).

Clinically, regarding preference, 40 patients (31.2%) were satisfied with bilateral TKAs without a specific preference, while 44 patients (34.3%) preferred TKAs of the G-ΔJLO knee and 44 patients (34.3%) preferred the S-ΔJLO knee (Figure 1). No significant difference was observed in the preference rates between the groups (*p* = 1.000). Additionally, no significant differences were found in the HSS, WOMAC, and ROM between the G- and S-ΔJLO knees at the last follow-up (Table 3).

No complications were reported in either the G- or S-ΔJLO knees during the follow-up period.

## 4. Discussion

The most important finding of the present study was that, within the predefined non-inferiority margin, greater JLO change was not inferior to smaller JLO change with respect to patient preference between paired bilateral PS TKAs at an average 3.9 years of follow-up.

Previous studies involving CR TKAs have shown that JLO preservation is a crucial factor in optimizing clinical outcomes and improving functional recovery. Winnock de Grave et al. [4] reported that JLO preservation resulted in gait pattern similar to those of healthy native knees without increasing knee adduction moments after CR TKA. Clark et al. [2] reported that robot-assisted CR TKAs with better JLO preservation achieved higher Forgotten Joint Score-12 scores and greater ROM than those with less JLO preservation.

However, other studies have challenged the advantages of JLO preservation in TKA designs that sacrifice the PCL. Recent gait analyses have shown that restoring the pre-arthritic joint line does not necessarily replicate native knee kinematics in TKA using a medially stabilized design with PCL sacrifice [6]. Rodriguez et al. [5] analyzed 23 pairs of bilateral paired TKAs with PCL sacrifice and found no significant difference in patient-reported outcomes between paired TKAs with and without CPAK type recreation accompanied by JLO restoration.

In the present study, substantial difference in the degree of JLO change was observed between paired bilateral PS-TKAs, despite using the same surgical technique. This may primarily be attributed to the inconsistent positioning of the intramedullary rod during femoral valgus resection, which is the inherent limitation of manual TKA [19,20]. In our procedure, the femur was resected first, and bony adjustments were prioritized to achieve gap balancing. An unintentional varus distal femur cut led to a compensatory valgus tibia cut, resulting in a JLO with a more proximal apex direction. Conversely, when the distal femur was resected in neutral alignment, varus tibia cut was used to achieve gap balancing (because most preoperative knees exhibit varus alignment), yielding a JLO with a more distal apex direction. Given that most knees had a distal apex JLO direction preoperatively, the latter scenario would result in less alteration of the JLO and better preservation of both the CPAK type and JLO direction.

Based on previous studies advocating for JLO preservation, inferior clinical outcomes would be expected in the G-ΔJLO knees, where the former scenario was implemented. However, the preference rate and patient reported outcomes of G-ΔJLO knees were not inferior to those of S-ΔJLO knees. Interestingly, 65.6% of patients (31.2% of patients were satisfied with bilateral TKAs without preference and 34.3% of patients preferred TKAs with greater JLO change) were satisfied with or preferred TKAs with greater JLO change.

Preserving the native anatomy may not play a critical role in the clinical outcomes of PS TKA sacrificing the PCL, which is an important structure contributing to native knee kinematics [7]. Song et al. [15] also reported comparable clinical outcomes, including patient preference between paired PS TKAs with an anterior tibial slope, which deviates significantly from the native anatomy, and those with a more anatomical PTS, after a minimum follow-up of 5 years. From this perspective, JLO preservation would not have a critical effect on the clinical outcomes of PS TKA.

Clinical outcomes after TKA are significantly affected by various personal factors [8,9,10,18]. A recent systematic review described personal factors, such as pain catastrophizing and psychological disorders, as key determinants of postoperative outcomes in TKA [18]. Therefore, evaluating paired PS TKAs in a manner that controls for personal factors will offer a clearer understanding of the clinical impact of JLO preservation. Based on our results, degree of JLO preservation may not be a dominant determinant of patient preference in PS TKAs. In light of these findings, the use of robots or navigation systems, which entail additional costs, to achieve better JLO preservation in PS TKA should be carefully considered [21].

This study has several limitations. First, this study is retrospective in design, as prospectively performing paired TKAs with markedly different JLO changes in a single patient would have been virtually impossible and ethically unacceptable. Second, although patients’ clinical outcomes usually stabilized in the second year after TKA, the follow-up period in this study was relatively short. This is because we have only recently adopted the current surgical technique in which bony adjustments are prioritized over soft tissue balancing to achieve gap balancing during TKA. Third, the >3° side-to-side difference in ΔJLO was adopted based on the previous literature, but it was not derived from a clearly established biomechanical or patient-perception threshold. Therefore, its direct clinical relevance in terms of patient awareness remains uncertain. Fourth, our operative procedure was performed based on mechanical alignment, which is not optimal for restoring native anatomy. Although S-ΔJLO knees showed better JLO preservation, neither group sufficiently reproduced the preoperative CPAK type and JLO direction, which may have contributed to the comparable clinical outcomes. Fifth, the present study did not evaluate changes in the JLO in the three dimensions. In addition to coronal alignment changes, alterations in the axial and sagittal planes may also affect postoperative outcomes. Sixth, although patient preference enables a unique within-subject comparison, it is inherently multifactorial. Not all potential confounders were fully controlled; for example, the influence of spinal or hip disorders on preference could not be precisely evaluated. Therefore, the absence of a significant difference in preference should not be interpreted as definitive evidence of clinical equivalence. Seventh, despite the assessment of various patient-reported outcomes, joint awareness-specific measures such as the Forgotten Knee Score were not evaluated. Finally, several factors should be taken into consideration when extrapolating the results of this study. Specifically, most patients were female because end-stage degenerative osteoarthritis is more prevalent among women in Asian populations [22,23]. In addition, most patients fell within the overweight range, but were not classified as obese. Moreover, all surgeries were performed by a highly experienced single surgeon at a tertiary care center.

## 5. Conclusions

The degree of joint line obliquity change was not associated with patient preference in bilateral PS TKAs. JLO preservation may not be a critical determinant of patient preference in PS TKA.

## Figures and Tables

**Table 1:** Patient demographics.

Operating Period	January 2018–December 2022
Number of patients	128
Age *	71.2 ± 5.0
Female/Male	120/8
Body mass index (kg/m^2^) *	26.1 ± 3.3
Type of prostheses (Attune^®^/ Persona^®^)	68/60
Follow-up period (year)	3.9 ± 1.5

* Continuous variables are presented as mean ± standard deviation.

**Table 2:** Radiographic results.

		G-ΔJLO	S-ΔJLO	*p*-Value
mLDFA (°)	Preoperative	89.4 ± 2.6	89.7 ± 2.2	0.148
	Postoperative	92.8 ± 1.7	90.8 ± 1.5	<0.001
mMPTA (°)	Preoperative	84.0 ± 2.5	84.0 ± 2.4	0.881
	Postoperative	91.1 ± 1.4	88.4 ± 1.5	<0.001
aHKA (°)	Preoperative	−5.4 ± 3.2	−5.6 ± 3.5	0.418
	Postoperative	−1.8 ± 1.8	−2.5 ± 2.3	0.002
	Change	3.6 ± 3.3	3.1 ± 3.3	0.261
JLO (°)	Preoperative	173.4 ± 3.9	173.7 ± 3.1	0.130
	Postoperative	183.9 ± 2.5	179.2 ± 2.1	<0.001
	Change	10.5 ± 3.3	5.5 ± 3.3	<0.001
PTS	Preoperative	10.7 ± 3.7	10.9 ± 3.3	0.199
	Postoperative	3.3 ± 2.3	3.4 ± 2.5	0.471
	Change	7.4 ± 4.4	7.5 ± 3.9	0.789

Data are presented as mean ± standard deviation. G-ΔJLO, TKAs with a greater change in the joint line obliquity; S-ΔJLO, TKAs with a smaller change in the joint line obliquity; mLDFA, mechanical lateral distal femoral angle; mMPTA, mechanical medial proximal tibial angle; aHKA, arithmetic hip–knee–ankle angle; JLO, joint line obliquity; PTS, posterior tibial slope.

**Table 3:** Clinical results.

		G-ΔJLO	S-ΔJLO	*p*-Value
HSS	Preoperative	37.1 ± 6.4	37.5 ± 7.6	0.249
	Last follow-up	93.2 ± 5.7	92.9 ± 5.0	0.540
WOMAC	Preoperative	68.2 ± 3.0	67.8 ± 4.0	0.155
	Last follow-up	9.8 ± 10.0	9.0 ± 8.8	0.307
Range of motion (°)	Preoperative	116.8 ± 17.2	118.3 ± 20.7	0.291
	Last follow-up	130.0 ± 15.1	128.8 ± 15.4	0.116

Data are presented as mean ± standard deviation. G-ΔJLO, TKAs with a greater change in the joint line obliquity; S-ΔJLO, TKAs with a smaller change in the joint line obliquity; HSS, Hospital for Special Surgery; WOMAC, the Western Ontario and McMaster Universities osteoarthritis index.

## Data Availability

The data that support the findings of this study are available on request from the corresponding author. The data are not publicly available due to privacy or ethical restrictions.
